# Toward robust clinical genome interpretation: Developing a consistent terminology to characterize Mendelian disease-gene relationships—allelic requirement, inheritance modes, and disease mechanisms

**DOI:** 10.1016/j.gim.2023.101029

**Published:** 2023-11-17

**Authors:** Angharad M. Roberts, Marina T. DiStefano, Erin Rooney Riggs, Katherine S. Josephs, Fowzan S. Alkuraya, Joanna Amberger, Mutaz Amin, Jonathan S. Berg, Fiona Cunningham, Karen Eilbeck, Helen V. Firth, Julia Foreman, Ada Hamosh, Eleanor Hay, Sarah Leigh, Christa L. Martin, Ellen M. McDonagh, Daniel Perrett, Erin M. Ramos, Peter N. Robinson, Ana Rath, David W. Sant, Zornitza Stark, Nicola Whiffin, Heidi L. Rehm, James S. Ware

**Affiliations:** 1 National Heart and Lung Institute and MRC London Institute of Medical Sciences, Imperial College London, London, United Kingdom;; 2 Dept of Medical Genetics, Great Ormond Street Hospital, Great Ormond Street, London, United Kingdom;; 3 Medical and Population Genetics, Broad Institute of MIT and Harvard, Cambridge, MA;; 4 Geisinger Autism and Developmental Medicine Institute, Danville, PA;; 5 Royal Brompton and Harefield Hospitals, Guy’s and St. Thomas’ NHS Foundation Trust, London, United Kingdom;; 6 Department of Translational Genomics, Center for Genomic Medicine, KFSHRC, Riyadh, Saudi Arabia;; 7 Department of Genetic Medicine, Johns Hopkins University School of Medicine, Baltimore, MD;; 8 INSERM, US14-Orphanet, Paris, France;; 9 Department of Genetics, University of North Carolina at Chapel Hill, Chapel Hill, NC;; 10 European Molecular Biology Laboratory, European Bio-informatics Institute, Wellcome Genome Campus, Hinxton, Cambridgeshire, United Kingdom;; 11 Department of Biomedical Informatics, University of Utah, Salt Lake City, UT;; 12 Dept of Medical Genetics, Cambridge University Hospitals, Cambridge, United Kingdom;; 13 Wellcome Sanger Institute, Wellcome Genome Campus, Hinxton, United Kingdom;; 14 Genomics England, Queen Mary University of London, Dawson Hall, London, United Kingdom;; 15 Geisinger Health System, Danville, PA;; 16 Open Targets, Open Targets, Wellcome Genome Campus, Hinxton, Cambridgeshire, United Kingdom;; 17 National Human Genome Research Institute, National Institutes of Health, Bethesda, MD;; 18 The Jackson Laboratory for Genomic Medicine, Farmington, CT;; 19 Australian Genomics, Melbourne 3052, Australia;; 20 Victorian Clinical Genetics Services, Murdoch Children’s Research Institute, Melbourne 3052, Australia;; 21 University of Melbourne, Melbourne 3052, Australia;; 22 Big Data Institute and Wellcome Centre for Human Genetics, University of Oxford, United Kingdom;; 23 Center for Genomic Medicine, Massachusetts General Hospital, Boston, MA

**Keywords:** Allelic requirement, Disease mechanisms, Gene curation, Inheritance modes, Ontology

## Abstract

**Purpose:**

The terminology used for gene-disease curation and variant annotation to describe inheritance, allelic requirement, and both sequence and functional consequences of a variant is currently not standardized. There is considerable discrepancy in the literature and across clinical variant reporting in the derivation and application of terms. Here, we standardize the terminology for the characterization of disease-gene relationships to facilitate harmonized global curation and to support variant classification within the ACMG/AMP framework.

**Methods:**

Terminology for inheritance, allelic requirement, and both structural and functional consequences of a variant used by Gene Curation Coalition members and partner organizations was collated and reviewed. Harmonized terminology with definitions and use examples was created, reviewed, and validated.

**Results:**

We present a standardized terminology to describe gene-disease relationships, and to support variant annotation. We demonstrate application of the terminology for classification of variation in the ACMG SF 2.0 genes recommended for reporting of secondary findings. Consensus terms were agreed and formalized in both Sequence Ontology (SO) and Human Phenotype Ontology (HPO) ontologies. Gene Curation Coalition member groups intend to use or map to these terms in their respective resources.

**Conclusion:**

The terminology standardization presented here will improve harmonization, facilitate the pooling of curation datasets across international curation efforts and, in turn, improve consistency in variant classification and genetic test interpretation.

## Introduction

The clinical application of genomic data is reliant upon a robust understanding of the relationships between locus, genotype, mechanism, and disease phenotypes.^1^

Assessment of the evidence that variants in a gene cause a particular monogenic disease is critical for variant classification, particularly for clinical application.^2–4^ The American College of Medical Genetics and Genomics and the Association for Molecular Pathology (ACMG and AMP) have issued standards and guidelines for the clinical interpretation of sequence variants, which have now been widely adopted internationally.^2^ These standards and further subsequent guidance make clear that the first step in the classification and interpretation of a variant is the robust assessment of disease-gene validity;^4^ without a clear understanding of the gene’s role in disease, variant assessment criteria cannot be accurately applied.^5,6^ Using an incorrectly classified variant for family cascade testing and the delivery of screening, treatment, or reproductive choices can have severe adverse consequences.

Historically, disease genes were identified by linkage studies in large families,^7^ often using polymorphic markers in close proximity to the gene responsible for the disease. Subsequently, candidate gene studies based on known or hypothesized disease mechanisms became commonplace.^8^ Until as recently as 10 years ago, it was not fully appreciated that individually rare genetic variants are collectively extremely common. Exome sequencing typically yields 200 rare (gnomAD allele frequency <0.1%) and a mean of 27 ± 13 novel (not present in population databases, number varies by ancestry) coding variants.^9^ Many studies reported new gene-disease associations without adequately controlling for background genetic variation in the population: inadequately small control cohorts, often just 100 chromosomes, were used for assessment of novel disease genes and variants, and consequently both the literature and disease databases were flooded with assertions of gene-disease relationships and variant pathogenicity that have not proven robust over time.^10–12^ Recent years have seen concerted efforts to correct this bias, using large, publicly available population databases, such as ExAC and later gnomAD,^13,14^ as control cohorts, and applying standardized approaches to reinterpret evidence for gene-disease relationships^5^ and variant pathogenicity^2^

Many groups are invested in curation of disease-gene validity, including academic and health care centers, private companies, and consortia. The Gene Curation Coalition (GenCC) is a coalition aiming to harmonize approaches among these entities to ensure gene-level curated resources are comparable and interoperable and to provide access to structured representations of consensus data. As a first undertaking, the GenCC developed a consensus term set for grading gene-disease validity and developed a unified database to display curated gene-disease validity assertions from its members (the Clinical Genome Resource [ClinGen], DECIPHER, Gene2Phenotype [G2P], Transforming Genetic Medicine Initiative, MedlinePlus Genetics, Genomics England PanelApp [PanelApp], PanelApp Australia, Online Mendelian Inheritance in Man [OMIM], Orphanet, Ambry Genetic, Illumina, Invitae, Mass General Brigham Laboratory for Molecular Medicine, Myriad Women’s Health, HUGO Gene Nomenclature Committee, Franklin by Genoox, King Faisal Specialist Hospital and Research Center, and PharmGKB). This database can be likened to a “ClinVar for genes” in that members can submit assertions of disease association but in this case for genes, not variants. The GenCC database provides a single route of access to comprehensive aggregated assertions (https://search.thegencc.org)^15^ and currently contains over 16,911 gene-disease assertions on 4704 unique genes from 12 submitters. OMIM will connect its large dataset to the GenCC in real time via a submission API being launched soon. Resolution of gene-disease validity discrepancy across GenCC submitters is ongoing using a manual review process.

As the next step to facilitate harmonized gene-disease validity assessments and support variant classification within the ACMG/AMP framework, the GenCC has focused on developing standardized terminology for the characterization of disease mode of inheritance, allelic requirement, and disease-associated variant consequences. Currently, groups utilize different terminology to describe these 3 characteristics. The considerable discrepancy in the derivation and application of these terms generates confusion and risks discordant assertions about pathogenicity of different classes of variants.

Although there is a close conceptual relationship between inheritance and allelic requirement, they are distinct and serve different purposes. Inheritance is used for describing the mode of transmission of a phenotype, eg, autosomal dominant and is particularly applicable in the clinical setting for communicating recurrence risk and to guide family screening and reproductive advice. Allelic requirement describes how many alleles must be affected to cause the relevant disease, eg, 1 allele (monoallelic) in dominant disease and both alleles (biallelic) in recessive disease. It is necessary for variant annotation pipelines and to determine if a given variant in a specific context is relevant to the phenotype of the patient, eg, a single heterozygous variant (in the absence of compound heterozygosity) may provide a diagnosis for dominant disease, but is insufficient to explain recessive disease, where a second contributory variant or alternative cause should be sought.

Disease-associated variant consequence, in particular, can be useful when evaluating novel variants in validated disease-associated genes. Is the predicted consequence of the novel variant consistent with that of previously reported pathogenic variants or with the mechanism of disease (if known)? For many genes, the mechanism of action will not yet be known even if the gene-disease validity has been confirmed. Understanding the consequence of known pathogenic variants is a useful intermediate step that can aid variant classification and inform understanding of the mechanism of disease. For example, the consequence of a nonsense-mediated-decay (NMD)-competent nonsense variant is a reduction in the amount of gene product produced. If all known pathogenic variants in a gene are nonsense, not only can we predict that a novel NMD-competent nonsense variant identified in a patient would have a high likelihood of being pathogenic, but we can also postulate that other novel variants with the same consequence (reduction in the amount of gene product produced) are likely to be pathogenic. Considering a NMD-competent nonsense variant, a whole-gene deletion or other variants resulting in premature termination codons (PTCs), including frameshift and essential splice site variants, could be considered equivalent, assuming they are located in required exons and sufficiently upstream to lead to lead to the same effect. Regulatory variants in non-coding regions that abolish protein expression can also have equivalent downstream effects.^16^ An advantage of using disease-associated variant consequence in an era of increasing appreciation of the clinical importance of non-coding variants is that it is applicable across both coding and non-coding region variants.

Having structured data representations compatible across platforms to describe inheritance, allelic requirement, and disease-associated variant consequence can help avoid duplication of effort, facilitate manual annotation, and can be more readily incorporated into automated analysis pipelines.

Many groups provide resources to disseminate gene-disease curations, with varying levels of detail, eg, some assess whether there is a gene-disease relationship, whereas others add details of disease mechanisms. Each group may use different terminology to describe inheritance (autosomal dominant, autosomal recessive, etc), allelic requirement, and both structural and functional consequences of a variant.^17–20^

The GenCC promotes use of standardized terms for structured representation of gene-disease relationships, including strength of evidence for the gene-disease association, disease mode of inheritance, allelic requirement, structural and functional consequences of genetic variation, and mechanism of pathogenicity. This will allow for harmonized terminology across genetic resources and aid variant curation, classification, and reporting.

## Materials and Methods

### Consensus development panel

**Figure 1:**
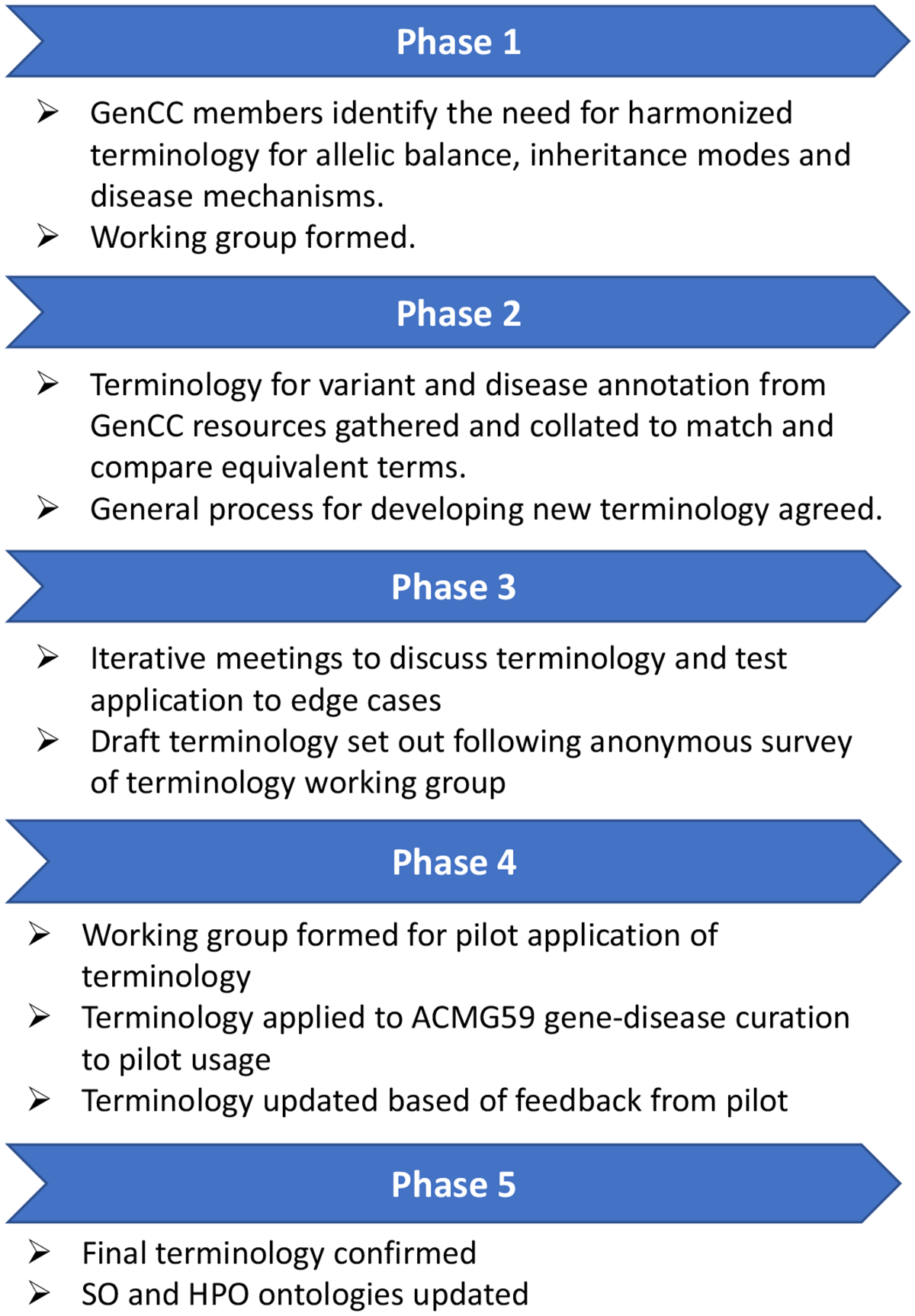
Process for developing and testing the terminology. HPO, human phenotype ontology; GenCC, The Gene Curation Coalition; SO, Sequence Ontology.

The process followed for developing and testing the terminology is outlined in Figure 1.

The GenCC includes experts in the identification and evaluation of variants from diverse settings, including clinical and research contexts, academic and commercial laboratories, software and resource developers, and organizations maintaining current nomenclature standards. An initial meeting and scoping exercise identified the need for a harmonized framework and terminology for inheritance, allelic requirement, and disease-associated variant consequences. The ultimate goal will be to understand precise mechanisms of disease and predict precise functional consequences of variants, but we have not yet developed a structured ontology for mechanism given the enormous diversity of possible functional effects of genetic variation. The groupings of predictable gene product changes will allow for consistent variant prioritization pending further functional characterization. The panel met by monthly conference call between February 2019 and September 2022. Existing terminology used by GenCC members and partners was reviewed and collated (Supplemental Tables 1 and 2). Existing terms for allelic requirement and inheritance coalesce around Human Phenotype Ontology (HPO) terms,^21^ whereas terms for disease-associated variant classification coalesce around Sequence Ontology (SO) terms.^22^

An updated framework and ontology were developed through iterative discussion and survey. All members of the panel reviewed and approved the final terminologies. Consensus was defined as agreement among most (>80%) members of the panel. The penultimate draft of the ontology and framework was generated following an anonymous online survey of the panel. Changes were made, as considered appropriate, based on feedback from the pilot working group.

### Pilot curation working group

A working group of clinical geneticists with experience in the identification and evaluation of variants in both the clinical and research settings was formed to pilot curation of the 59 genes (66 disease-gene pairs) included in the ACMG Recommendations for Reporting of Incidental Findings in Clinical Exome and Genome Sequencing version 2.0^23^ (current at the time of study) using the new terminology and variant consequence matrix. The templates supplied to curators in the pilot working group are available in Supplemental Information 1 & 2, and the final outputs are available online (github.com/ImperialCardioGenetics/ACMGSF_pilot_curation/).

## Results

### Terminology for inheritance and allelic requirement

Most coalition members used separate inheritance and allelic requirement terms, with the exception of G2P, Genomics England PanelApp, and PanelApp Australia, which use only allelic requirement terms.

### Inheritance

Review of existing inheritance terms identified substantial consistency in high-level terms used, eg, autosomal recessive/autosomal dominant, with the exception of G2P, Genomics England PanelApp and PanelApp Australia, which use monoallelic/biallelic as stem terms for both inheritance and allelic requirement (see Supplemental Table 1). Some groups also used qualifier terms, which can be applied to some or all stem terms to add further granularity, for example, indicating whether a gene is imprinted, the penetrance of variation in a gene, or whether pathogenic variants in a given gene are typically de novo or mosaic. There was less consistency between GenCC members in whether qualifiers were used and in qualifier terms themselves. Most consortium members’ existing stem terminology mapped broadly to HPO inheritance terms. It was also noted that there were also some redundant HPO inheritance terms (Supplemental Table 3).

It was agreed to collaborate with HPO to update HPO inheritance terms (children of HP:0000005), and to harmonize on the first level. HPO inheritance terms are a relatively small ontology intended to describe the mode of inheritance and contain terms such as “Autosomal dominant inheritance HP:0000006.” Original HPO inheritance terms also included several terms that were not true modes of inheritance but did provide useful genetic information, such as “Genetic anticipation HP:0003743” (Supplemental Table 3). Three new subcategories of Mode of Inheritance HP:0000005 were created (Table 1). Mendelian Inheritance (HP:0034345) retains only true inheritance terms (Table 2), whereas Inheritance qualifier (HP:0034335) was coined to record descriptors and optional specifications such as “Displays anticipation” or “Typically de novo” that provide relevant, useful information and can be used in conjunction with any inheritance term (Table 3), and non-Mendelian inheritance HP:0001426 captures non-Mendelian disease ranging from digenic to complex disease due to multiple major and minor genetic determinants possibly together with environmental factors. HPO Mendelian inheritance terms were extended to capture all required information for our purposes, for example, adding new terms to describe genes encoded in pseudoautosomal regions (PAR recessive HP:0034341, PAR dominant HP:0034340). Following rigorous review, iterative discussion, and survey, a final list of inheritance terms and qualifiers was agreed upon (Tables 2 and 3).

### Allelic requirement

Consistency was also identified between GenCC groups in the use of existing allelic requirement terms, with most groups using monoallelic/biallelic as stem terms, with the exception of DECIPHER, which used zygosity terms for variants, eg, “heterozygous” (see Supplemental Table 2). Again, there was less consistency between members in the use of qualifiers, ranging from no qualifiers to structured qualifier terms to long narrative qualifiers. HPO did not previously have terms for allelic requirement; therefore, a proposed terminology was derived from first principles and terms most commonly used among GenCC members. Following iterative discussion and survey, a final list of allelic requirement terms was agreed upon that captures whether the disease results from monoallelic or biallelic variation, whether encoded on an autosome, a sex chromosome, or the pseudoautosomal region, and for monoallelic diseases on the X chromosome whether they manifest when heterozygous or hemizygous.

### Alignment of inheritance and allelic requirement terms

Finalized terms for inheritance and allelic requirement, which had been derived independently of each other, were aligned and adjusted as needed such that each mode of inheritance had an accompanying allelic requirement term to describe the context necessary to cause disease (Table 2). Allelic requirement terms were added to HPO as allelic requirement synonyms of Mendelian inheritance terms and share HPO identifiers with the corresponding inheritance term. Cross-cutting inheritance qualifiers generated for use with inheritance terms above were expanded by consideration of edge cases such as craniofrontonasal dysplasia due to *EFNB1*, which requires heterozygosity.^24^ Qualifier terms were refined to be compatible with all inheritance and allelic requirement terms by iterative discussion, to enable recording of data important to reproductive advice and family screening. Examples to illustrate the applications of these terms are shown in Box 1.^25,26^

Terms are specific to each disease-gene pair. Considering a hypothetical example of a gene on the X chromosome in which biallelic or hemizygous monoallelic variation causes congenital structural heart abnormalities, but a heterozygous monoallelic variant typically presents with late onset cardiomyopathy, this might be coded as monoallelic_X_hemizygous for congenital heart disease and appropriate filtering applied in a developmental disorders panel for diagnosis of an infant and monoallelic_X_heterozygous (Typified by age-related onset) for cardiomyopathy, with different variant filtering applied for a cardiac gene panel analysis in an adult. This scenario is an example in which genetic variation in a single gene can give rise to separate disease phenotypes that are curated as distinct entities and has the advantage of tracing the evidence for each disease association.

### Variant classes and disease-associated variant consequences

Structured terminology to capture disease-associated variant classes (missense, nonsense, etc) is required to support variant classification within the ACMG/AMP framework, both to infer which variant classes are likely to be relevant, and to support assessment of relevance of functional evidence.

Terminology is required for both disease-associated variant classes (eg, missense variant) and the disease-associated consequence of those variants (eg, altered gene product sequence). There may be good evidence for a particular variant class causing disease without a good understanding of the disease mechanism. For example, for *MYH7* and cardiomyopathy, which is typically caused by missense variants and, less frequently, splice variants, but not by PTCs, one might establish that disease requires the presence of an abnormal gene product, and the gene is not haploinsufficient, long before determining exactly how the beta myosin heavy-chain protein function is perturbed (activating variants via loss of certain interactions, loss of ability to enter low energy relaxed state, gain of ATPase activity, etc), or which particular perturbations lead to clinically distinct types of cardiomyopathy (hypertrophic cardiomyopathy vs dilated cardiomyopathy).

The 2015 ACMG/AMP sequence variant interpretation guideline provided a framework for classifying variants based on several evidence criteria indicative of benign or pathogenic features, including a criterion (PVS1) specific to predicted loss-of-function variants. PVS1 is defined as “null variant (nonsense, frameshift, canonical ±1 or 2 splice sites, initiation codon, single or multi-exon deletion) in a gene where loss of function (LOF) is a known mechanism of disease.” This is a somewhat oversimplified functional interpretation of a specific set of sequence variants. Although these variant classes are, indeed, most likely to cause NMD and effectively be null or lead to LOF because of a truncated product, there is also potential for gain of function through loss of a regulatory region (either terminal in the case of a truncation or internal in the case of an in-phase deletion) or action as a dominant negative or poison peptide. Furthermore, a missense variant, not included in the list above, also has potential to act as loss-of-function variant, gain-of-function variant or as a dominant negative variant. The functional (and thus clinical) consequences of a given variant are only partially predictable from sequence alone. As such, it is necessary to describe which predictable consequences have been associated with disease-gene pair and/or are consistent with a known molecular mechanism of disease, if we are to apply appropriate filters in a variant prioritization pipeline.

For a novel or previously uncharacterized variant, we usually have understanding of sequence consequence, eg, amino acid sequence, but not (directly) the functional effect, which speaks to mechanism. The ability to capture high-level predictable consequences (disease-associated variant consequence) when the precise functional effect is unknown would be beneficial. For example, a nonsense variant could lead to “decreased gene product level,” and a missense variant could lead to an “altered gene product sequence” (altered amino acid sequence). Similarly, recognizing the high-level consequences of known pathogenic variants for a disease-gene pair reported in the literature or in ClinVar can help predict both mechanism and other variant classes that may have similar consequences. For example, if nonsense variants are pathogenic, we might expect other variants leading to “decreased gene product level,” such as frameshift variants, to have similar effects, even if not previously observed. The weight given to PVS1 in the ACMG/AMP framework highlights the importance of correctly identifying disease-associated variant consequences.

The terminology presented here is intended to be compatible with Ensembl Variant Effect Predictor (VEP) and other variant annotation tools that use SO terms for consequence. The SO is a structured, controlled vocabulary for the definition of sequence features used in biological sequence (eg, DNA, RNA, or peptide) annotation.^22^ There are ~200 SO terms for variant consequence including a mix of terms to describe variant class (eg, missense_variant SO:0001583) and variant function (eg, gain_-of_function_variant SO:0002053), with a subset of 33 variant classes used by Ensembl VEP and 42 by SnpEff,^27^ whereas Annovar^28^ outputs 19 variant classes but does not use SO terms.^29^ Ensembl VEP requires outputs to be calculable based on sequence alone.

It was agreed to collaborate with SO to update the 33 variant consequence terms used by VEP. Redundant SO terms (eg, downstream_gene_variant) were culled and new terms added to capture the different impact of variants either triggering or escaping NMD (Table 4).

**Figure 2:**
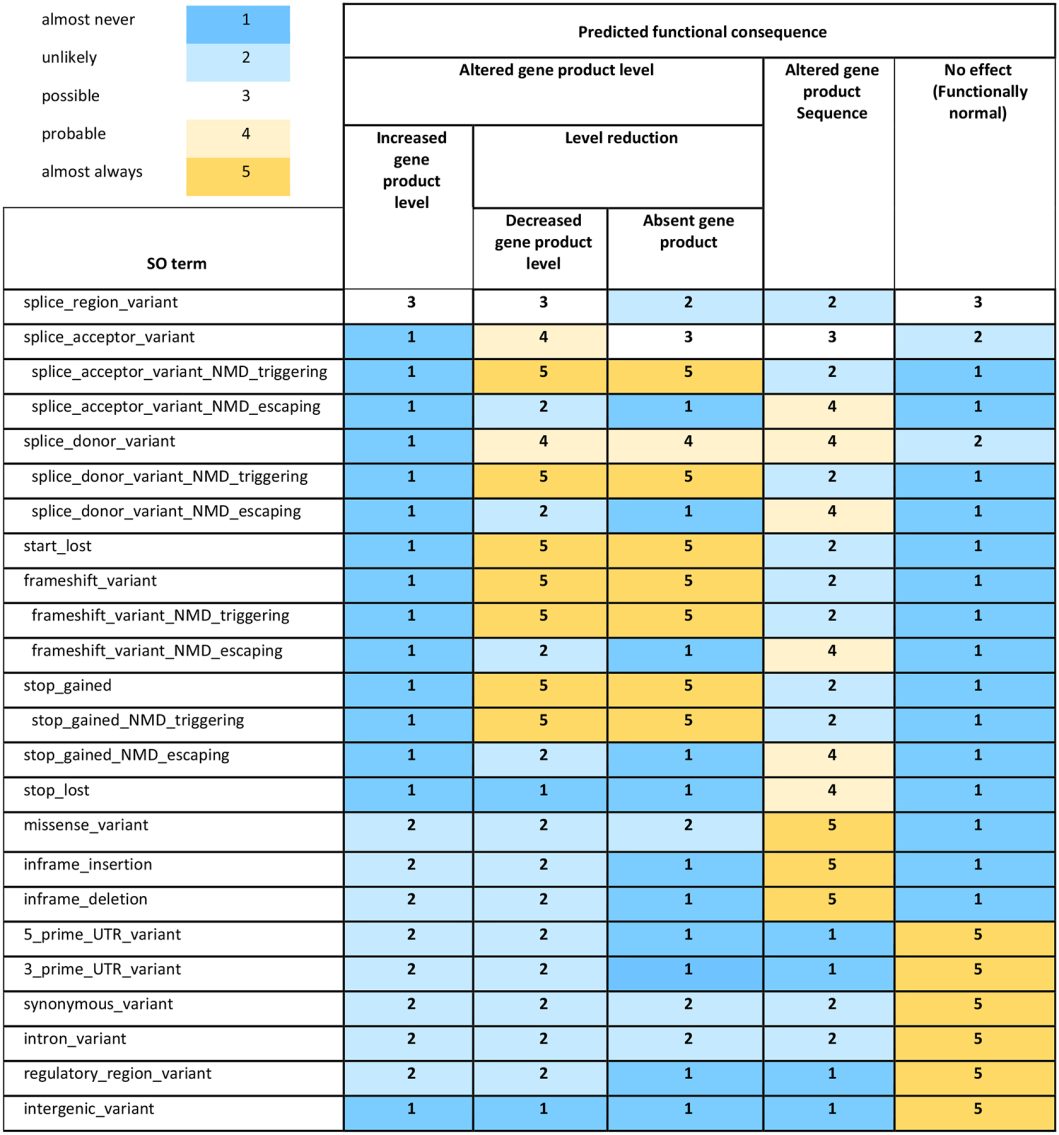
Matrix of 6 new high-level predicted functional consequences mapped to SO structural consequence terms via a semi-quantitative scale indicating likelihood of each high-level consequence. The semi-quantitative scale is characterized from first principles by expert evaluation. NMD, nonsense-mediated-decay; UTR, untranslated regions; SO, Sequence Ontology.

High-level terms to describe predictable variant consequences (disease-associated variant consequence terms) were proposed (Table 4) and trialed by mapping consequence terms to SO variant class terms. Following iterative discussion and survey, a matrix was generated mapping 6 high-level predictable disease-associated variant consequence terms (altered gene product level, decreased gene product level, absent gene product, increased gene product level, altered gene product sequence, and functionally normal) to more specific variant classes described by SO variant class terms (Figure 2). These are mapped via a semi-quantitative scale representing the likelihood of each consequence (1: almost never, 2: unlikely, 3: possible, 4: probable, 5: almost always), characterized from first principles by expert evaluation.

In brief, different classes of variant may alter the level (abundance) or the sequence of the gene product or may have no effect on either. A gene, for our purpose, is a segment of DNA that encodes an RNA that, in turn, performs some function; the product of a coding gene is a protein, and the product of a non-coding gene is non-coding RNA. For variants that alter gene product level, the direction of effect may be known (increased or decreased/absent) or unknown (altered). “Decreased gene product level,” therefore, captures the group of variant classes typically analyzed together because they often result in a PTC, ie, NMD-competent nonsense and frameshift variants, as well as most variants at canonical splice donor/acceptor sites—also sometimes described in the literature as (predicted) protein truncating variants (PTVs), or loss-of-function variants (although missense variants can also result in decreased or absent protein levels through a variety of mechanisms). Metrics such as constraint have improved understanding of the impact of other, non-PTC variants, such as promoter or untranslated region variants, on the level of gene product produced, and these could also be captured by “Decreased gene product level.”

“Altered gene product sequence” captures sequence-altering variants such as missense, NMD-incompetent PTCs, and other length-changing variants (eg, inframe indels and stop loss) that would change the amino acid sequence of a protein or the nucleotide sequence of a non-coding RNA. Multiple disease-associated variant consequence terms can be linked to a given variant class. For example, a splice donor variant may lead a “decreased gene product level” and/or an “altered gene product sequence” (altered amino acid sequence due to altered splicing).

When gathering evidence for a new disease-gene relationship, it is likely that the types of variants associated with disease may be determined before their precise mechanistic consequences are understood. For example, a new gene-disease relationship may be curated with evidence that the disease-associated variant classes are those leading to “altered gene product sequence,” without knowing whether these lead to LOF (eg, through mis-folding, loss of an active site, or altered trafficking), or gain of function (increased enzyme activity or ion channel conductance, or new poison peptide activity). The more information available about a variant (eg, computational, transcriptomic, or functional studies), the more specific one can be with the functional consequence term, for example, a 5′ untranslated region variant could lead to either increased or decreased expression, therefore, in the absence of more specific knowledge, the “altered gene product level” term could be applied, but following expression studies, this could be manually updated to either “increased gene product level” or “decreased gene product level.”

### Results from pilot study

We piloted the new terminology for inheritance, allelic requirement, and disease mechanism using the ACMG SF v2.0, 59 genes (66 gene-disease pairs).^23^ We generated curations for the purpose of piloting the terminology, and these curations are not intended for application to clinical practice. The list includes genes related to inherited cariovascular disease, cancer phenotypes, and inborn errors of metabolism, and the gene-disease pairs are characterized by a range of inheritance types and different disease mechanisms. All 66 gene-disease pairs were successfully described using the new terminology (github.com/ImperialCardioGenetics/ACMGSF_pilot_curation/). Note that these are informal curations for the purpose of this study only; for official curations, please see the ClinGen website https://clinicalgenome.org/.

Several changes were suggested and implemented after the pilot (see Supplemental Information 1 for the initial draft terminology for comparison with final terminology in Tables 2 and 3 and Supplemental Information 2). The inheritance qualifier “Typified by age-related onset” was added to allow description of conditions in which age of onset is typically later in life, ie, adulthood, and in which penetrance is dependent on the age of the subject. This was found to be particularly relevant for the cancer susceptibility and inherited cardiovascular condition genes included in the pilot. HPO Onset terms (children of HP:0003674) can be used to provide granularity at the case level where that information is known. The need to capture information regarding structural variants, as well as sequence variants was also noted, and SO terms will be developed for these as a separate initiative.

Based on our experience of undertaking the pilot curations and incorporating the finalized terminology, we have developed a suggested template for curation using this framework (see Supplemental Information 2). In Box 2, we show condensed examples of gene-disease pair curation using this framework.

## Discussion

With the increasing availability of DNA sequencing, application in clinical practice has become routine, including increasingly comprehensive sequencing with larger panels of genes, exome, or genome sequencing.^30,31^ Consequently, the number of variants identified in patients undergoing genetic testing is increasing. Curation of gene-disease relationships and of inheritance pattern, allelic requirement, pathogenic variant classes, and disease- associated variant consequence are vital for the efficient and accurate classification of variants and effective clinical application of genetic information.^6,17,32^

Streamlined filtering systems that reduce the number of extraneous variants (ie, benign or non-actionable) for a laboratory to manually review are of increasing importance with the convergence of routine genomic sequencing and guidelines for the reporting of secondary findings.^33^ The current responsibility for interpreting the clinical relevance and actionability of a variant falls to the reporting laboratory and, ultimately, the clinician. Some laboratories report all variants, including variants of uncertain significance (VUS), whereas others only report pathogenic or likely pathogenic results. Secondary findings, by definition, are much more likely to fall outside the area of expertise of the reporting laboratory or clinician. For example, an oncologist or cancer geneticist may order exome or genome sequencing from a specialist cancer genetics laboratory for their patient with suspected hereditary cancer, but sequencing may identify a reportable secondary finding in a cardiovascular gene, such as *KCNQ1* causing Long QT syndrome or *FBN1* causing Marfan Syndrome, and vice versa. Robust, structured data on disease mechanisms are key to accurate interpretation of potential significance.

We successfully engaged a diverse group of experts to establish consensus standard terms and a systematized approach for mode of inheritance, allelic requirement, and disease-associated variant consequence and present these as a structured resource. The final terms presented in Figure 2 will be used by members of the GenCC in sharing gene curations and when filtering variants for disease-relevant variant consequences. We suggest these terms may provide a standard terminology across diverse areas of clinical genetics, including clinical genetic laboratory reporting and gene-disease curation efforts.

This structured resource can be used by individual laboratories or curation programs alongside variant filtering pipelines. After assessment of which variant classes are consistent with a disease using this framework, pipelines can then be adjusted to prioritize only those relevant classes.

Use of these standardized terms will facilitate the assessment of which variant classes are likely to be disease-relevant (eg, to apply PVS1), as well as the evaluation of functional evidence to determine which ACMG/AMP rules are applicable for a variant under interpretation. These terms will also be relevant for the next version of the ACMG/AMP sequence variant classifications standards under development (personal communication, H. Rehm). In addition to streamlining disease-gene curation and facilitating interpretation of variants in established disease-gene pairs, this standardized terminology will aid in assessment of novel potential disease-gene or disease-variant relationships. For a previously unseen variant, we usually understand the genetic consequence but not (directly) the functional effect. Similarly, we may not know the precise mechanism for a disease or variant but can interpret likely disease-relevant variant classes.

One aim of the terminology is to aid interpretation of novel variants based on established pathogenic variant classes for a given gene-disease pair. In our pilot, curators were asked to test this utility by matching reported variant classes to high-level disease-associated variant consequence terms in the matrix (Figure 2) and identifying additional variant classes that were likely to have the same disease-associated variant consequence. For example, if most known pathogenic variants for disease A were “stop gained” (aka nonsense), which has a likelihood score of “5: almost always” for “decreased gene product level” and “absent gene product,” our pilot curators could then infer that a novel variant of a class that also has a high likelihood (4: probable or 5: almost always) for “decreased gene product level” or “absent gene product” (eg, an NMD competent frameshift), could reasonably be included in filters for relevant variants. Additional variant classes identified in this way as potentially pathogenic will be dependent on the initial pathogenic variant classes identified.

During our pilot, it was noted that the output of informal curations of inheritance, allelic requirement, disease-associated variant consequence, and predicted pathogenic variant class can be curator-dependent. Early formative feedback from our working group revealed that variant classes recorded as pathogenic for a given disease-gene pair can be dependent on how exhaustive a literature search was undertaken and the level of evidence each curator required in order to assign pathogenicity. We improved internal guidance to provide clarity regarding the evidence requirement for pathogenicity, and curations were updated to confirm consistency. Our pilot working group was formed of clinical geneticists experienced in clinical variant interpretation and application of ACMG guidelines but not formal curation efforts. This highlights the importance of standardized guidance setting out clear criteria for gene-disease association and variant pathogenicity applied in formal curation efforts.^5,34^ Standardizing curation methods is beyond the scope of this project that instead focuses on providing a terminology framework for future work to be built upon. Our ontology has since been successfully applied for the curation of inherited cardiovascular condition gene-disease pairs, presented as a structured dataset and available publicly as the CardiacG2P.^35^

For some gene-disease pairs, there are well established, often recurrent, pathogenic variants that are the only pathogenic variant in that gene, or that are rare and atypical causes of disease. For example, almost all evidence for *KCNQ1* as a cause of short QT syndrome is derived from a single missense variant (NP_000209.2:p.(Val141Met)) via a gain-of-function mechanism,^36^ but this does not by itself inform whether other missense variants may cause disease or if LOF is also a possible mechanism of disease. Although the proposed terminology provides a robust framework, additional structured data will be needed to fully represent the repertoire of disease-associated variation for any given gene.

For some disease-gene pairs, such as malignant hyperthermia (*CACNA1S* and *RYR1*), the mechanism (eg, dominant negative or haploinsufficiency) is not clear.^37^ If there is only limited evidence for haploinsufficiency, one should not assume that all variant classes predicted to reduce gene product would be pathogenic. The decision whether to retain only variants with a high likelihood of pathogenicity, or all variants that could plausibly have an effect consistent with pathogenesis of a particular disease, will depend on the desired sensitivity and specificity of a genomic interpretation pipeline.

Craniofrontonasal dysplasia due to *EFNB1*, an X-linked condition that does not manifest (fully) if hemizygous,^24^ is an example of an edge case that required the introduction of a specific cross-cutting inheritance qualifier (requires heterozygosity HP:0034343). Every effort has been made to identify other edge cases through regular meetings of the consensus development panel and the terminology pilot; however, it may become apparent that additional terms are necessary after this framework is more broadly applied.

Duchenne and Becker muscular dystrophy due to variants in dystrophin were also explored as an edge case as complex rearrangements in the gene are often encountered.^26^ Approximately 60% of pathogenic dystrophin variants are large insertions or deletions that lead to frameshift errors downstream, whereas approximately 40% are substitutions or small insertions and deletions. Because of the structure and function of the protein, even a large in-frame deletion may result in the milder Becker phenotype if the N and C termini of the protein remain intact. In addition, for many of the cancer predisposition genes, whole gene and other large deletions and structural rearrangements can cause disease. Together these cases highlight the need for an ontology to describe structural variants, which is currently under development as a separate GenCC initiative.

In summary, correctly classifying variants is of utmost importance for management of genetic conditions, including for family cascade testing and the delivery of screening and treatment services, as well as supporting reproductive choices. Curation of gene-disease validity and of mode of inheritance, allelic requirement, and disease-associated variant consequences are vital to support accurate variant classification. Currently, groups, including academic and health care centers, private companies, and consortia, utilize different terminology to describe these 3 characteristics. The considerable discrepancy in the derivation and application of these terms generates confusion and risks discordant assertions about pathogenicity. The GenCC promotes use of standardized terms for structured representation of gene-disease relationships, including strength of evidence for the gene-disease association, disease mode of inheritance, allelic requirement, structural and functional consequences of genetic variation, and mechanism of pathogenicity. Here, we propose consensus terminology to aid in the characterization of gene-disease relationships. This will allow for harmonization across genetic resources, and aid variant curation, classification, and reporting.

## Supplementary Material

Supplementary Material

## Figures and Tables

**Table 1:** New hierarchy of HPO mode of inheritance child terms and definitions

HPO Term	Definition
**Mode of inheritance**. HP:0000005	The pattern in which a particular genetic trait or disorder is passed from one generation to the next.
Mendelian inheritance HP:0034345	A mode of inheritance of diseases whose pathophysiology can be traced back to deleterious variants in a single gene. The inheritance patterns of these single-gene (monogenic) diseases based on the work of Gregor Mendel.
Inheritance qualifier HP:0034335	The terms in this hierarchy can be used to specify the context in which inheritance of a disease is typically observed.
Non-Mendelian inheritance HP:0001426	A mode of inheritance that depends on genetic determinants in more than one gene.

*HPO*, human phenotype ontology.

**Table 2:** Harmonized allelic requirement and Mendelian inheritance terms, child terms of HP:0034345

Allelic Requirement Term	Inheritance Term	HPO ID
monoallelic_autosomal	Autosomal Dominant	HP:0000006
biallelic_autosomal	Autosomal Recessive	HP:0000007
monoallelic_X_heterozygous	X-linked Dominant	HP:0001423
monoallelic_X_hemizygous	X-linked Recessive	HP:0001419
monoallelic_Y_hemizygous	Y-linked	HP:0001450
mitochondrial	Mitochondrial	HP:0001427
monoallelic_PAR	PAR dominant	HP:0034340
biallelic_PAR	PAR recessive	HP:0034341

*HPO*, human phenotype ontology; *PAR*, pseudoautosomal region.

**Table 3:** Inheritance qualifier terms- these optional terms can be combined with either inheritance terms or allelic requirement terms to provide additional information about the relationship of a disease-gene pair. Penetrance describes the proportion of genotype-positive individuals that develop disease given a lifespan of 80 years. Expressivity describes the severity and scope of phenotypic manifestation

Inheritance Qualifier HP:0034335 (Parent and Child Terms)	Definition (Parent Term)
Typified by somatic mosaicism HP:0001442	Description of conditions in which affected individuals typically display somatic mosaicism, ie, genetically distinct populations of somatic cells in a given organism caused by DNA variants, epigenetic alterations of DNA, chromosomal abnormalities, or the spontaneous reversion of inherited variants. In many conditions typified by somatic mosaicism, constitutive variants are lethal, and cases are exclusively or predominantly mosaic.
Typically de novo HP:0025352	Description of conditions that are exclusively or predominantly observed because of de novo variants. In some cases, this may be due to the limited reproductive fitness of affected individuals.
Typified by incomplete penetrance HP:0003829 - Typified by moderate penetrance HP:4000159 - Typified by high penetrance HP:4000158	Description of conditions in which not all individuals with a given genotype exhibit the disease. Penetrance is the proportion that develop disease given a lifespan of 80 years. Examples include, *CYP1B1* glaucoma, which has approximately 90% penetrance; Van der Woude syndrome due to *IRF6* causes cleft lip and/or palate with penetrance estimated at 80%; *C9orf72* causes frontotemporal dementia and/or amyotrophic lateral sclerosis with approximately 50% penetrance. Typified by moderate penetrance HP:4000159 Description of conditions in which only a moderate proportion of individuals with a given genotype exhibit the disease regardless of age assuming a full lifespan of 80 years. There is no commonly accepted definition for moderate penetrance, but we suggest that this term be applied if at least 20 percent, but less than 80 percent of individuals with the given genotype would manifest the disease with a full lifespan. Typified by high-penetrance HP:4000158 Description of conditions in which only an incomplete but relatively high proportion of individuals with a given genotype exhibit the disease regardless of age assuming a full lifespan of 80 years. There is no commonly accepted definition for incomplete but high penetrance, but we suggest that this term be applied if at least 80 percent but less than 100 percent of individuals with the given genotype would manifest the disease with a full lifespan.
Typified by complete penetrance HP:0034950	Description of conditions in which all individuals with a given genotype exhibit the disease within a lifespan of 80 years. For example, penetrance of Neurofibromatosis type 1 due to *NF1* is close to 100%. Penetrance describes the proportion of genotype positive individuals that develop disease given a lifespan of 80 years.
Typified by highly variable age of onset HP:0034857	Description of conditions in which age of onset is highly variable even in family members who share the same disease-associated variant or variants.
Typified by age-related onset HP:0003831	Description of conditions in which age of onset is typically later in life and in which penetrance is dependent on the age of the subject Additional terms to capture details of age of onset at an individual level are available within HPO as child terms of Onset HP:0003674.
Imprinted HP:0034338 - With maternal imprinting HP:0012275 - With paternal imprinting - HP:0012274	Requires that the abnormal allele be paternal or maternal in origin, depending on the disease-gene relationship. Imprinting refers to a normal developmental process in which either the paternal or maternal allele is inactivated, depending on the specific locus, thus leading to expression from only one copy of the gene. Disease typically manifests when a deleterious variant is inherited from a parent whose copy of the gene would normally be expressed, but not when a deleterious variant is inherited from a parent whose copy of the gene would normally be inactivated.
Displays anticipation HP:0003743	A phenomenon in which the severity of a disorder increases, or the age of onset decreases, as the disorder is passed from one generation to the next, typically due to expansion of a repeat sequence. For example, myotonic dystrophy is caused by triplet repeat expansion in the *DMPK* gene.
Requires heterozygosity HP:0034343	Covers rare instances of a condition that is most severe in the heterozygous state. Such disorders are rare and currently all are X-linked. Most X-linked recessive conditions manifest if hemizygous in males, or biallelic in females, though may have a mild phenotype in the heterozygous state in females. However, Craniofrontonasal dysplasia due to *EFNB1*, and *PCDH19*-related epilepsy, are both X-linked dominant and paradoxically more severe in females. Hemizygous males may be mildly affected but seldom manifest the full phenotype. Importantly the mutant allele can be inherited from a normal or very mildly affected father. The mechanism is currently accepted to be due to cellular interference whereby the 2 distinct cell populations (those with and without the variant) exhibit abnormal cellular interactions in the mosaic state—in women, who are functionally mosaic due to random X inactivation, or mosaic males. The same mechanism could theoretically be observed in autosomal genes with a mosaic variant.
Sex-limited expression HP:0001470 - Male-limited expression HP:0001475 - Female- limited expression - HP:0034344	Condition in which the phenotype only manifests in 1 sex, ie, either manifests in males or females but not both. Example: autosomal recessive sex reversal due to *DHH* on chr12 manifests only in XY males causing gonadal dysgenesis, whereas XX females are phenotypically normal.
Contiguous gene syndrome HP:0001466	Syndrome caused by the effects of abnormality (typically a deletion or duplication) of 2 or more adjacent genes.

*HPO*, human phenotype ontology.

**Table 4:** Hierarchy of SO disease-associated variant consequence terms

SO Hierarchy
sequence_variant SO:0001060
functional_effect_variant SO:0001536
altered_gene_product_level SO:0002314
decreased_gene_product_level SO:0002316
absent_gene_product SO:0002317
increased_gene_product_level SO:0002315
altered_gene_product_sequence SO:0002318
function_uncertain_variant SO:0002220
functionally_abnormal SO: 0002218
functionally normal SO:0002219
**Definitions of high-level terms with examples**
**Altered gene product level** - A sequence variant that alters the level or amount of gene product produced. This high-level term can be applied where the direction of level change (increased vs decreased gene product level) is unknown or not confirmed, eg, promoter or enhancer variants, some splice variants
**Increased gene product level** - a variant that increases the level or amount of gene product produced, eg, non-disruptive gene duplications, some promoter or enhancer variants
**Decreased gene product level** - a sequence variant that decreases the level or amount of gene product produced, eg, a 5′ UTR variant that reduced protein levels by disrupting translation, a 3′ UTR variant that affects RNA stability, splice variants that decrease but do not stop expression, variants leading to nonsense-mediated-decay (NMD)-competent premature termination codon (PTCs), or gene-disrupting structural variants.
**Absent gene product** - a sequence variant that results in no gene product. eg, whole gene or other large scale disruptive structural variant, variants producing NMD-competent PTCs
**Altered gene product sequence** - a sequence variant that alters the sequence of a gene product. eg, missense variants, NMD-incompetent PTCs, and other length-changing variants (in-frame indels, stop loss) Downstream mechanisms are then diverse: functionally null - misfolded, mislocalized, inactive, hypomorphic; disruptive presence of abnormal protein (gain-of-function (GoF), dominant negative) etc.
**Functionally normal** - a sequence variant that is not expected to alter gene product sequence or levels eg, a synonymous variant

New high-level terms shown in blue.

*SO*, Sequence Ontology; *UTR*, untranslated region.

## Data Availability

Data presented in this paper are publicly available in the supplementary materials and online (github.com/ImperialCardioGenetics/ACMGSF_pilot_curation/).

## Box 1. Example applications of harmonized allelic requirement terms

An X-linked dominant condition would be curated as monoallelic_X_heterozygous and we would understand that those diseases will manifest in heterozygous females and hemizygous males. Disease or affected males may be rarely observed if hemizygosity of the variant allele is extremely severe or lethal. Similarly, homozygous/compound heterozygous females may be seen with more severe manifestations.

eg, *Rett syndrome due to MECP2, a severe neurodevelopmental disorder that occurs almost exclusively in females*.^25^ *Rarely, classically affected males with somatic mosaicism or an extra X chromosome have been described, usually with an earlier onset of symptoms*.

An X-linked recessive condition would be curated as monoallelic_X_hemizygous and we would understand that those diseases are most commonly observed in males and may not fully manifest when heterozygous in females. Although they can manifest with ameliorated phenotype or manifest if skewed inactivation, etc., we intend that this is implicit in the term as characteristic of many sex-linked disorders and do not anticipate that an additional qualifier term is needed to communicate this, unless the heterozygous phenotype is sufficiently distinct as to be classified as a different disease entity. It is also implicit that monoallelic variants in females with chromosomal anomalies (eg, 45,X) and biallelic variants in females would also meet the allelic requirement.

eg, Duchenne muscular dystrophy (DMD).^26^ Males are affected by childhood onset skeletal muscular dystrophy and later onset cardiomyopathy, typically in adolescence. Approximately 10% of female heterozygotes have some (typically mild) symptoms and 20% have cardiac involvement on investigation. Heterozygote females and females with biallelic variants with the full DMD phenotype have been reported.

## Box 2. Condensed examples of gene-disease curation using the finalized terminology and template

**Table:**

Gene	*SCN5A*
OMIM gene number	600163
Disease name	*SCN5A*-related long QT Syndrome
Gene disease validity (ClinGen)	DEFINITIVE
Inheritance	Autosomal dominant
Allelic requirement	Monoallelic autosomal
Inheritance qualifiers	Typified by incomplete penetrance
Disease-associated variant consequence	Altered gene product sequence
Variant classes reported with evidence of pathogenicity	Missense; inframe insertion; inframe deletion
Potential novel variant classes based on predicted functional consequence	splice_acceptor_variant_NMD_escaping; splice_donor_variant_NMD_escaping; splice_donor_variant; frameshift_variant_NMD_escaping; stop_gained_NMD_escaping; stop_lost
**Narrative summary** Altered gene product sequence of *SCN5A* causes long QT syndrome. The likely disease mechanism is gain of function. Over 200 pathogenic missense variants and in-frame deletions or insertions have been reported. It is thought that gain-of-function *SCN5A* pathogenic variants lead to enhanced sodium current, which can trigger life-threating arrhythmias. Rare missense variants are estimated to occur in around 2% of healthy White and 5% of healthy non-White subjects so collectively missense variants are not rare in the healthy population. It has been noted that approximately 10% of genotype positive LQT patients have more than 1 mutation in >=1 gene. Biallelic pathogenic variants or digenic pathogenic variants appear to be generally associated with a more severe phenotype with longer QTc interval and a higher incidence of cardiac events. Note: loss of function variants in *SCN5A* are associated with Brugada syndrome and individual variants can have hybrid loss of function and gain of function effects causing a mixed phenotype.	
Gene	*MYBPC3*
OMIM gene number	600958
Disease name	*MYBPC3*-related Hypertrophic Cardiomyopathy
Gene disease validity (ClinGen)	DEFINITIVE
Inheritance	Autosomal dominant
Allelic requirement	Monoallelic autosomal
Inheritance qualifiers	Typified by incomplete penetrance
Disease-associated variant consequence	Decreased gene product level; altered gene product sequence
Variant classes reported with evidence of pathogenicity	Splice_region; splice_acceptor; splice_donor; frameshift; frameshift_variant_NMD_triggering; stop_gained; stop_gained_NMD_triggering; missense; inframe_insertion; inframe_deletion; intron_variant; structural_variants (whole exon deletions)
Potential novel variant classes based on predicted functional consequence	splice_acceptor_variant_NMD_escaping; splice_donor_variant_NMD_escaping; frameshift_variant_NMD_escaping; stop_gained_NMD_escaping; stop_lost; start_lost
**Narrative summary** *MYBPC3* pathogenic variants cause HCM through decreased gene product level or altered gene product sequence either leading to a reduction in MyBP-C content in the sarcomere or altered function. The disease mechanism is loss of function; There is evidence of haploinsufficiency. Inheritance is usually autosomal dominant, typified by incomplete penetrance and variable expressivity. Homozygous and compound heterozygous variants have been reported and can lead to severe, early onset phenotypes. The majority of variants are heterozygous frameshift, nonsense, or splice site variants that result in premature termination codons. Missense and inframe indels are also frequently reported and a subset have been shown to cause loss of function through failure of myofilament incorporation and rapid degradation, further supporting haploinsufficiency as a mechanism. Variants in *MYBPC3* affecting canonical splice site dinucleotides are a well-characterised cause of HCM. Recent work has identified more deeply intronic variants associated with disease.	
